# Detection and localization of viral infection in the pancreas of patients with type 1 diabetes using short fluorescently-labelled oligonucleotide probes

**DOI:** 10.18632/oncotarget.14896

**Published:** 2017-01-29

**Authors:** Niels Busse, Federico Paroni, Sarah J. Richardson, Jutta E. Laiho, Maarit Oikarinen, Gun Frisk, Heikki Hyöty, Eelco de Koning, Noel G. Morgan, Kathrin Maedler

**Affiliations:** ^1^ Islet Biology Laboratory, University of Bremen, Germany; ^2^ Islet Biology Exeter, University of Exeter Medical School, UK; ^3^ Department of Virology, School of Medicine, University of Tampere, Tampere, Finland; ^4^ Department of Immunology, Genetics and Pathology, Uppsala University, Sweden; ^5^ Fimlab Laboratories, Pirkanmaa Hospital District, Tampere, Finland; ^6^ Department of Internal Medicine, Leiden University Medical Center, Leiden, The Netherlands; ^7^ Hubrecht Institute/University Medical Center Utrecht, Utrecht, The Netherlands

**Keywords:** enteroviruses, type 1 diabetes, pancreas, islets, oligonucleotide probes, Pathology Section

## Abstract

Enteroviruses, specifically of the Coxsackie B virus family, have been implicated in triggering islet autoimmunity and type 1 diabetes, but their presence in pancreata of patients with diabetes has not been fully confirmed.

To detect the presence of very low copies of the virus genome in tissue samples from T1D patients, we designed a panel of fluorescently labeled oligonucleotide probes, each of 17-22 nucleotides in length with a unique sequence to specifically bind to the enteroviral genome of the picornaviridae family.

With these probes enteroviral RNA was detected with high sensitivity and specificity in infected cells and tissues, including in FFPE pancreas sections from patients with T1D. Detection was not impeded by variations in sample processing and storage thereby overcoming the potential limitations of fragmented RNA. Co-staining of small RNA probes in parallel with classical immunstaining enabled virus detection in a cell-specific manner and more sensitively than by viral protein.

## INTRODUCTION

Type 1 diabetes (T1D) is a chronic multifaceted disorder that results from selective autoimmune-mediated destruction of the insulin producing β-cells. Environmental factors [1], together with genetic predisposition [2], interact cooperatively to initiate chronic islet autoimmunity [3].

Viruses have been proposed as possible initiators of islet autoimmunity and were first implicated as long ago as the nineteenth century although it was not until much later that a clear association was established between mumps and diabetes [4–6]. Improvements in molecular biology subsequently broadened the panel of viruses which are implicated in causing diabetes [7, 8] and the weight of evidence now suggests that coxsackieviruses [9] play a role. In support of this, a clear correlation between enterovirus infection and the onset of T1D was revealed in association studies [10] and via a comprehensive meta-analysis [11].

Coxsackieviruses belong to the *Picornaviridiae*, and are small positive-sense single stranded RNA viruses, which have been shown recently to induce a persistent, slowly-replicating infection in both myocardium and pancreas. This may result from alteration to the viral genome during the progress of infection including the generation of naturally occurring 5′-deletions [12–14]. Several direct (immunohistochemistry) and indirect (serology, isolation of viruses from patients) approaches have confirmed the presence of enterovirus both in the circulation and in the islets of T1D patients [15–20]. Enteroviruses in the pancreas were detected by immunostaining for viral protein (VP1) [17, 21], which is highly expressed under acute viral infection and diminishes in persistent infection and may not be detected under circumstances where viral replication is compromised [22]. Also, non-specific interaction of the VP1 antibody with other cellular proteins has been reported [23, 24].

A well-characterized cohort of human pancreatic donor tissue has been established by nPOD (Network for Pancreatic Organ Donors with Diabetes) and is available, similar to other cohorts, mainly as formalin fixed, paraffin embedded samples (FFPE) [25]. This method ensures preservation of the samples for many years, but a limiting factor is the relatively poor RNA integrity often associated with FFPE preservation which means that analysis of tissue samples by PCR can be difficult [26]. Also, detection of the viral genome in the pancreas has been challenging and more sensitive and reliable methods are required.

This has created pressure to develop alternative (accurate and equally sensitive) methods to allow the detection of RNA in single cells within FFPE tissue samples. An obvious candidate is *in situ* hybridization, where labeled oligonucleotide probes specifically pair with target nucleic acids via Watson-Crick base pairing and some success has been gained using long probes (~50-100nts) labeled with either radioisotopes or enzymes catalyzing chromogenic reactions. However, this approach also has limitations in the face of samples with poor RNA integrity or very low target RNA copy numbers. Thus, improved RNA FISH methods have been developed to overcome these hurdles. Enteroviral RNA was detected by a new generation of RNA probes (QuantiGene^®^ ViewRNA Assay), which depend on signal amplification [25]. This approach offers the advantage of signal amplification via the use of branched secondary probes but, theoretically, may be affected by several conditions. To create a docking site for the branched probes, two probes must sit adjacent to one-another on the target sequence, effectively lengthening the de facto short probes. However, RNA degradation can still affect the docking of probes and hence signal enhancement. Flexibility in choosing the binding site with the best thermodynamic characteristics may also be compromised.

Because of such limitations to the detection of enteroviruses with high accuracy and sensitivity, we present here an adapted method to target single RNA molecules with short (~20nts) fluorescently labeled oligonucleotides *in situ*. These oligonucleotides anneal to common regions of the RNA genome of members of the coxsackievirus family. Such short singly labeled oligo RNA probes are resistant to RNAse and RNA detection is less affected by target RNA degradation, making these probes more versatile while retaining sensitivity and specificity. Because their binding to the target sequences occurs independently for each probe, this gives probe combinations the advantage that there is a degree of freedom in their positioning without risk of the loss of stringency, efficiency or specificity.

To generate a distinct fluorescent signal above background noise, a sufficient amount of labeled probes must bind in close proximity [27–29], thereby ensuring high specificity and considerable flexibility of detection after hybridisation. The use of small contiguous RNA species also overcomes the potential limitation of fragmented target RNA. With our newly established protocol, we successfully detect viral RNA in both cell culture and FFPE tissue sections, in combination with classical immunostaining. Short probes were able to detect viral infection at lower viral loads than classical immunostaining and the method is comparable in sensitivity to that of semi-nested PCR [30].

## RESULTS

### An established protocol for short RNA-oligoprobe labeling in FFPE tissue sections

**Figure 1:**
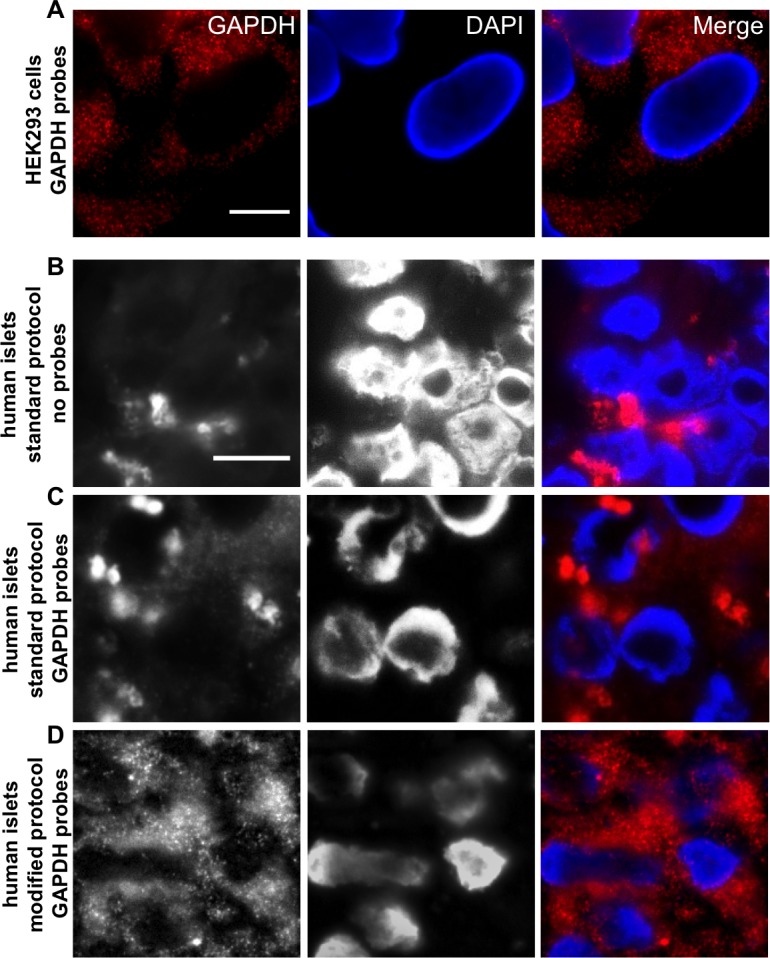
An established protocol for short RNA-oligoprobe labeling in FFPE tissue sections **A**. GAPDH oligonucleotides were tested in the cell line HEK 293. Single fluorescent spots, each representing one single RNA molecule, are clearly visible. **B**. Normal deparaffinization procedure shows high background noise in FFPE islet sections even without probes (No Probes) and does not allow clear distinction of probe signal and background, when GAPDH oligonucleotides were added **C**.; Standard Protocol. Modification of the deparaffinization and post-hybridisation protocol leads to background reduction and increased signal intensity of GAPDH oligonucleotides **D**.; Modified Protocol. RNA Probes are labeled with Quasar 570 (red) and nuclei were stained with DAPI (blue), scale bar depicts 10µm.

In order to develop a robust protocol for RNA-oligoprobe labeling of FFPE pancreatic tissue sections, we initially tested a commercially available probe set targeting the housekeeping gene (GAPDH) in cultured HEK293 cells and in isolated FFPE human islets (Figure 1). Natural, as well as fixative-derived signal noise, is a major problem when employing fluorescence microscopy to detect probes targeting RNA molecules, especially when these are present in low abundance. Thus, while our test probes gave a very specific signal in cultured HEK293 cells (Figure 1A), probing of FFPE human islets generated high background noise both in the absence of probes (Figure 1B) and with the GAPDH probes (Figure 1C), when following the standard protocol. Reduction in the FFPE-derived background signal was achieved by the removal of any remaining paraffin wax crystals (see material and methods). This involved the use of a protocol in which xylene washes were undertaken at high temperatures prior to a step utilizing pepsin/HCl to separate proteins from nucleic acids. In addition, Sudan black was included to reduce the overall FFPE-derived background fluorescence. Using this modified protocol, single positive dots (representing hybridization to as few as one RNA molecule) could be detected within the Abbe-diffraction limit and were easily discriminated from any residual background noise (Figure 1D).

### RNA-oligonucleotide probe design and specificity

**Figure 2:**
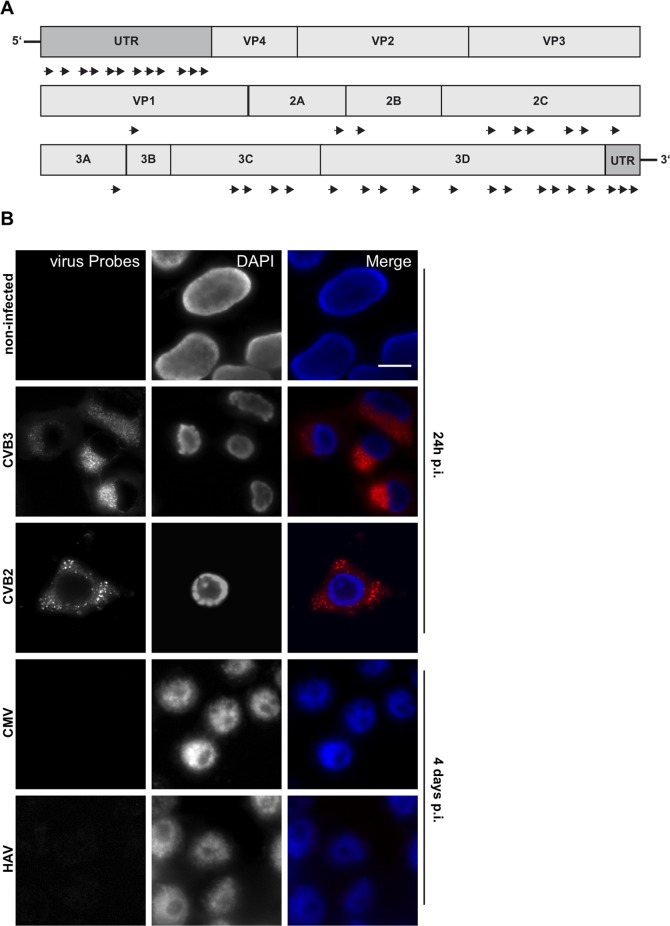
RNA-oligonucleotide probe design and specificity **A**. Scheme of custom-designed oligonucleotide (CVB_1) annealing throughout the viral genome. **B**. Viral RNA probes were tested against non-infected, CVB3 and CVB2 (100% and 79% similarity to consensus sequence, respectively; positive control), CMV, (DNA virus; negative control) and HAV (<45% similarity to consensus sequence) infected HEK 293 cells. Cells were infected with an MOI of 5 and harvested after 24h (control, CVB3, CVB2) or 4 days (CMV, HAV) post-infection. RNA Probes are labeled with Quasar 570 (red) and nuclei were stained with DAPI (blue), scale bar depicts 10µm.

Probe set CVB_1 included a wide range of group B coxsackieviruses; it consisted of a mixture of 40 short oligonucleotides, each comprising 17-22 nucleotides covering the whole viral genome (see material and methods and Figure 2A). This enabled us to target single RNA molecules. To detect a positive signal, it was determined that at least 17 of the probes in a given set must bind to their target sequence with only one mismatch allowed with respect to the stringency parameters [25].

To test the specificity of the probe set CVB_1, HEK293 cells were fixed and processed after either culture without viruses or following infection with coxsackieviruses CVB3 and CVB2 (Figure 2B) which share a sequence similarity of about 79%. Cells were infected for 2h at an MOI of 5 and the viruses allowed to replicate for 24h prior to fixation and analysis. Probes efficiently detected viral genomes within infected cells (Figure 2B, CVB3 and CVB2) whereas signal was absent from uninfected cells (Figure 2B, non-infected), confirming that the virus-specific probe set had no off-target effects.

The specificity of the probe set was further tested in HEK293 cells infected with cytomegalovirus (CMV), a DNA virus of the *herpesviridae* family (Figure 2B, CMV) or hepatitis A virus (HAV), a positive ssRNA virus of the *picornaviridae* family (Figure 2B, HAV). Following infection at an MOI of 5 and incubation for 4 days to ensure viral replication, there was no visible cytopathic effect. The presence of virus was confirmed by RT-PCR (Suppl.Figure 1A). CMV appeared not to be in an active phase of replication as no signal was generated by RT-PCR of DNAse-treated samples. In neither HAV nor CMV infected cells were probe-specific hybridization spots detected, confirming the probe specificity. HAV shares partial sequence similarity with CVB3 (<45%) while CMV is a DNA virus; for each, the number of “on target” probes was below the detection limit of the assay.

The likelihood that the signals detected in samples were non-specific was further excluded by staining CVB3 infected islets in the absence of the probe set and by staining uninfected cells with the probe set. In each case, the negative controls delivered no staining, whereas virally infected cells yielded positive signals (Suppl.Figure 1B). RNAse A treatment abolished the signal from infected cells and confirmed that the probe set is specific to viral RNA (Suppl.Figure 1B).

We further tested the CVB_1 probe set on an array of cell lines previously generated for use with Quantigene^®^ ViewRNA virus probes [21]. Green monkey kidney cells (GMK and Vero), the human cervix (HeLa), alveolar (A549) epithelial carcinomic and rhabdomyosarcoma (RD) muscle cells were infected with viruses from the enterovirus groups A and B or adenovirus (DNA virus) [21] (Table 1 and Suppl.Figure 2). In line with the results obtained with Quantigene^®^ ViewRNA by Laiho et al. [21] the CVB_1 probe set yielded positive staining for viruses of both groups A and B, while it did not stain cells infected with adenovirus and human parechovirus 1 (HPeV1) (0/ 40 probes binding). Also, there was no binding to sequences from coxsackievirus A5 (11/40 probes theoretically match the virus sequence).

**Table 1:** RNA oligonucleotide staining of different picornaviridae and control viruses

Virus	Strain	Result	Virus	Strain	Result
**EV71**	PB-EV71Hus	**++**	**Echo3**	PB-E3DiT23	**++**
**CVB1**	ATCC	**++**	**Echo4**	ATCC	**++**
**CVB2**	ATCC	**++**	**Echo6**	ATCC	**+**
**CVB3**	ATCC	**++**	**Echo9**	ATCC	**+**
**CVB4**	ATCC	**++**	**Echo11**	ATCC	**+**
**CVB5**	ATCC	**++**	**Echo30**	ATCC	**++**
**CVB6**	ATCC	**++**	**PV3**	Sabin	**+**
**CVA2**	PB-CVA2V38	**++**	**HPeV1**	ATCC	**-**
**CVA4**	PB-CVA4V36	**++**	**Adenovirus C**	VR846	**-**
**CVA5**	PB-CVA5V43	**-**	**A549 cells**	-	**-**
**CVA6**	PB-CVA6V303V	**+**	**RD cells**	-	**-**
**CVA9**	ATCC	**++**	**Vero cells**	-	**-**
**CVA10**	PB-CVA10V2530	**++**	**HeLa cells**	-	**-**
**CVA16**	PB-CVA16V55	**++**	**GMK cells**	-	**-**

Probe specificity was tested on a cell array (FFPE); different cell lines were spotted either as uninfected controls or infected with different viruses. The results obtained with RNA probes set CVB_1 are displayed. Representative images are shown in Fig. S2.

### RNA-oligonucleotide probe sensitivity and consistency

**Figure 3:**
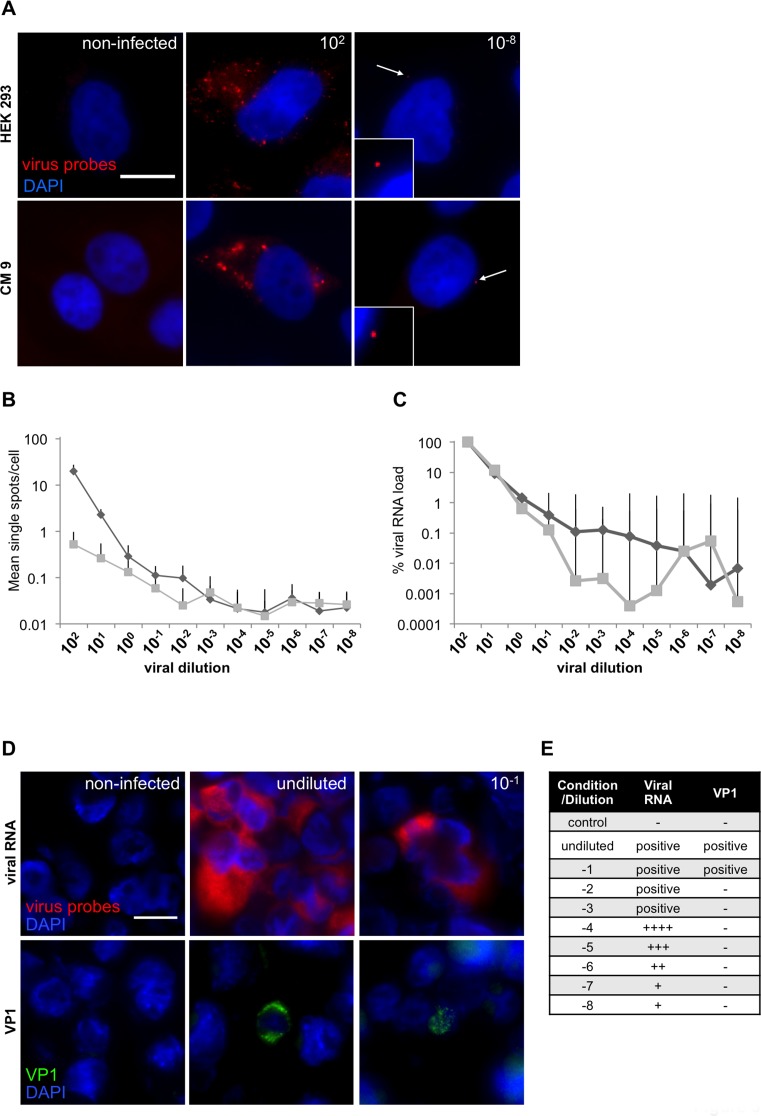
RNA-oligonucleotide probe sensitivity **A**. CM 9 and HEK 293 were infected with a dilution series (MOI 10^2^-10^-8^) of CVB3 and stained with custom-designed oligonucleotides (CVB_1). Representative images of CM9 and HEK293 cells infected with either the highest or lowest dilution of CVB3 of the series are shown. White arrows highlight single viral spots. **B**. Ten single images were acquired for each dilution and single fluorescent spots were manually counted. Results for HEK 293 (diamonds) and CM 9 (squares) are displayed as single spots per cell in logarithmic scale. In total, viral particles were counted in 4470 HEK293 cells and CM9 cells. **C**. Viral RNA from a parallel experiment was extracted and analyzed by PCR; MOI of 100 (10^2^) was set as 100%. **D**. Viral RNA (red) and VP1 (1/2000; green) staining on a CVB1-dilution array of FFPE infected GMK cells mixed with uninfected cells. RNA Probes were labeled with Quasar 570 (red) and nuclei were stained with DAPI (blue), scale bar depicts 10µm. **E**. Summary of the viral RNA and VP1 signals obtained from a CVB1-dilution array of FFPE infected GMK cells.

Next, we evaluated sensitivity of the CVB_1 probe set. Our RNA-FISH system was compared with two of the most widely used and well established techniques for virus detection: RT-PCR and immunohistochemistry using an antibody against the viral capsid protein 1 (VP1). RT-PCR is the most powerful and specific tool for RNA detection; but RNA accessibility and degradation are two important limiting factors. To compare the sensitivity of the RNA probes with analysis by PCR, the pancreatic line CM9 [31] and HEK293 cells were infected with CVB3. Since both RT-PCR and RNA-FISH can, in principle, detect the presence of a single RNA molecule, we used successive 10-fold serial dilutions of virus prior to infection of cells and to compare the sensitivity of the methods. Cells were plated in duplicates and the virus (CVB3; starting MOI of 100) was centrifugally inoculated at 16^°^C for 1h to synchronize the infection. After inoculation, any unbound viruses were removed by washing and the cells incubated at 37^°^C for an additional hour, to allow virus internalization and genome release from the capsid, before fixation or cell lysis for RNA extraction. Both RNA-FISH (Figure 3A, 3B and Suppl.Table 1) and RT-PCR (Figure 3C and Suppl.Table 2) were able to detect the presence of viral genomes, even at the highest dilution (10^-8^) (Figure 3B, 3C). Using RNA-FISH probes, single RNA molecules were detected and a plateau reached at a dilution of 10^-3^ virus by both visual counting and RT-PCR (Figure 3B-3C). The specificity of the RT-PCR results were confirmed by examination of dissociation curves, which were identical in all cases while samples from non-infected cells showed no signal (data not shown).

We next compared the efficiency of the CVB_1 probe set with the widely used VP1 (clone 5-D8/1, Dako cytomations) antibody, using a cell array of the human alveolar basal epithelial cell line A549 infected with CVB1 for 2, 4 and 6 h to generate a population representing different stages of infection [27]. After infection, cells were serially diluted with uninfected A549 cells to achieve a range from undiluted to 10^-8^. The cells were then fixed and paraffin embedded. When employed at a dilution of 1:2000 (which ensures specificity and minimizes the possibility of false positives [23, 24]), the VP1 antibody yielded positive signals only at dilutions of 10^-1^ or lower, whereas the RNA-FISH probes were able to detect viral RNA even at the highest dilution of 10^-8^ (Figure 3D, 3E).

The design of the RNA-FISH system should circumvent the problem of RNA degradation since it employs multiple probes to detect the target RNA. RNA fragmentation frequently occurs in, for example, autopsy samples, where processing and storage under RNAse free conditions is unlikely. We therefore tested FFPE CVB1-infected GMK cell sections, which had been infected and processed at the same time, but then cut and processed at different times (covering periods between 2012 and 2015) and stored at room temperature. The probes showed similar signals in all three samples regardless of the processing and storage time. Importantly, uninfected controls were negative (Suppl.Figure 3).

### Coupling RNA-FISH and immunohistochemistry

Important advantages of the use of RNA-FISH probes relate not only to their high sensitivity and specificity but also their ability to localize RNA molecules within specific cells of tissues such as the pancreas. We, therefore, investigated the localization and distribution of the signal emanating from the viral probes within the pancreas and compared this with detection by immunohistochemistry with the widely used VP1 antibody.

**Figure 4:**
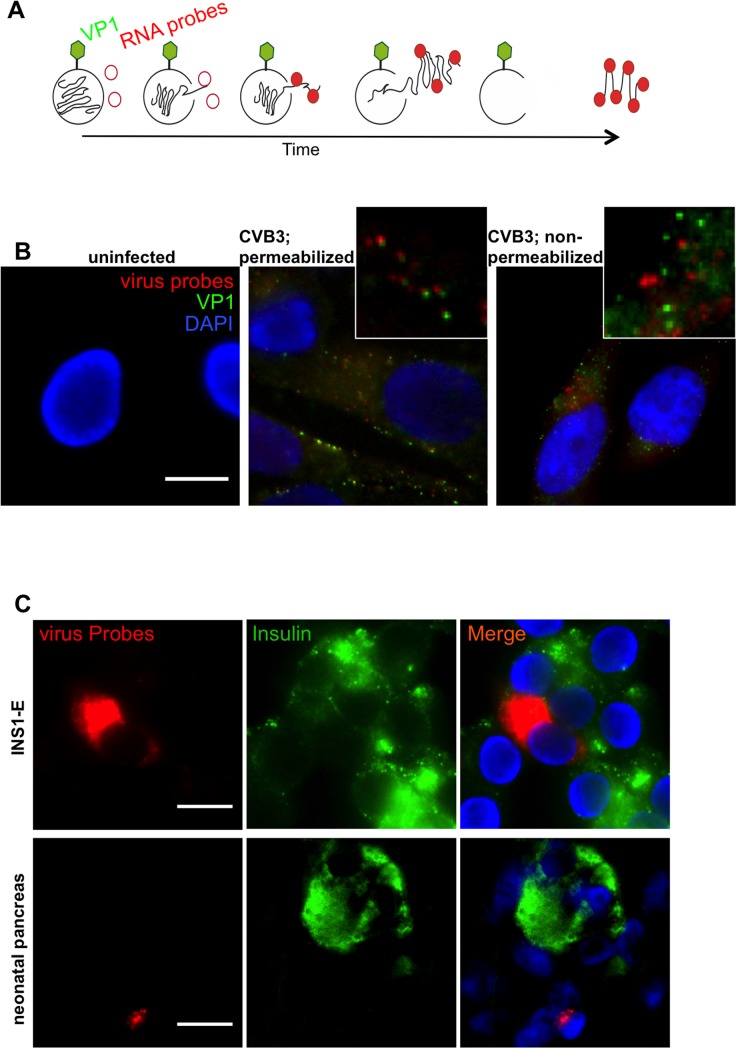
Coupling RNA-FISH and Immunohistochemistry **A**. Theoretical scheme of viral RNA and VP1 co-staining. Initially, labeled oligonucleotides cannot bind viral RNA within the capsid, but only when the virus is released. On the other hand, VP1 antibody can bind to the capsid surface. Over time as more RNA is released, more probes can anneal to their target sequence. When a sufficient amount of oligonucleotides is bound, green (VP1) and red (RNA) signal are visible in close proximity. **B**. CM9 cells were infected with CVB3 (MOI 1000) for 1h at 16°C, fixed and probed for viral RNA and VP1. VP1 (green) was found in close proximity with viral RNA (red), when capsid and RNA were present in the cytosol area (B; middle panel). When VP1 staining was performed before cell permeabilization no colocalization of viral RNA/VP1 was detected (B; right panel). No cross-reactivity was found with cellular proteins or RNAs (B; left panel). **C**. Co-staining and localization of viral RNA (red) and insulin (green) in CVB3-infected INS-1E cells and HgCl_2_ fixed paraffin embedded CVB-infected neonatal pancreas. Samples were stained with RNA FISH probes first, analyzed and then stained for insulin. The non-granular insulin staining in this slide appeared also by classical single insulin immunohistochemistry and is caused by the tissue condition. Nuclei were visualized by DAPI staining (blue); scale bar depicts 10µm.

Figure 4A shows a schematic representation of the expected profile of virus-staining: RNA probes (red) are not expected to anneal to the viral RNA while it is packaged within the capsid. However, once released within the cell, the probes should bind. Conversely, the VP1 antibody (green) should always bind to the capsid surface or, conceivably, to free VP1 which has not been incorporated into capsids. To visualize any differential labeling, human CM9 cells were infected with centrifugally inoculated CVB3 at an MOI of 1000. (Figure 4B). Anti-VP1 was detected at the plasma membrane and in the cytoplasm while RNA probes were localized exclusively in the cytoplasmic area (Figure 4B, middle panel and Suppl.Figure 4 for enhanced signals). As depicted at larger magnification, signals from the RNA molecules co-localized, or were in close proximity with those arising from the capsid protein, but the two were not superimposed. To verify the staining patterns, we also performed VP1 immunohistochemistry prior to cell permeabilization and subsequent RNA-FISH, so that the signals should not co-localize. As shown in Figure 4B (right panel), under these conditions the signals arising from each method of detection were clearly separate. Uninfected CM9 cells were used as controls and showed no nonspecific signal for either anti-VP1 or the RNA-probes (Figure 4B).

**Figure 5:**
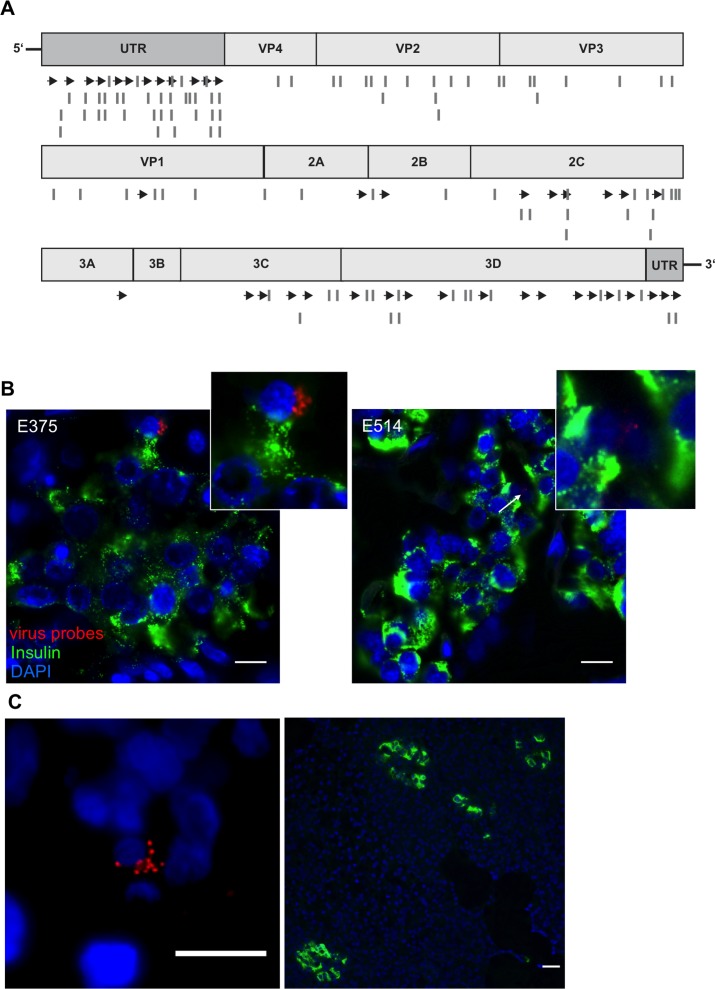
Viral RNA within an islet of a T1D patient **A**. The original probe set CVB_1 was complemented with two additional sets (CVB_2 and CVB_3). Scheme of custom-designed probe sets annealing throughout the viral genome. Arrows show localization of CVB_1, lines show the positions of CVB_2 and CVB_3 probe sets. **B**., **C**. Representative images of donors E375 and E514 form the UK cohort and of donor 3626 from the nPOD cohort. Virus RNA was found within the endocrine area (B) and outside the islets (C) shown by the co-staining of viral RNA probes (combination of CVB_1, CVB_2 and CVB3) (red) and insulin (Green). Tissues were first probed for viral RNA, analyzed and then stained for insulin. Nuclei were visualized by DAPI staining (blue); scale bar depicts 10µm.

Successful double staining of viral RNA and VP1 was confirmed in FFPE infected mouse spleen. Spleen sections from mice infected with CVB1 were stained with the CV_1 probe set to detect the viral genome and subsequently with anti-VP1 in an additional round of staining of the same section. Both techniques showed positive staining within the same regions of the samples (Suppl.Figure 5A).

Double staining of FFPE CVB3 infected cultured rat INS1-E cells for insulin and virus probes showed co-localization of viral RNA within insulin positive INS-1E cells (Figure 4C). Close proximity of viral RNA and insulin was also found in a Coxsackie infected neonatal mouse pancreas (Figure 4C), confirming the established double-staining protocol also in HgCl_2_ fixed and paraffin embedded cells. To demonstrate efficacy for virus staining in various tissues, we stained a section from a coxsackie-infected neonatal heart and found cells displaying a strong signal corresponding to viral RNA (Suppl.Figure 5B).

### Viral RNA localization in the pancreas in T1D

Having established the validity of our approach, we then used an enterovirus genome alignment (Suppl.Figure 6) to design two additional probe sets (CVB_2 and CVB_3; Suppl.Tables 4,5), which would complement the first set and allow detection of the entire range of group B enteroviruses. A scheme of the binding positions of the newly designed probe sets is shown in Figure 5A. We then used a combination of the three probe sets for a blinded analysis of autopsy pancreatic tissue recovered from nondiabetic and T1D patients having remaining residual β-cells from a UK cohort [31, 32]. As shown in Table 2, we were able to detect the presence of enteroviruses in 7 of 8 pancreas samples from T1D patients and in 2 of 8 nondiabetic controls. While viral RNA was found within the insulin-positive islet area in 6 of 8 T1D patients (Table 2 and Figure 5B), 5 of 8 pancreata also yielded positive signals for enteroviral RNA in the exocrine area. A single T1D patient from the UK collection had viral RNA exclusively in the exocrine area of the pancreas and not in the islets (although in this patient, only 1 fragmented and 2 normal islets were found throughout the whole section). Similarly, this distribution of viral RNA was also observed in a pancreas transplant biopsy from a patient with T1D who had developed recurrent disease from the nPOD-Transplantation cohort [32] (Figure 5C). In one nondiabetic control, we also found viral RNA in islets. Comparison of our results with anti-VP1 staining on the same samples performed separately in Exeter, 66% concordance was achieved. Thus, while virus was detected using viral probes in 6 out of 8 T1D pancreata within the insulin-positive islet area, VP1 staining was present in only 4/8 pancreata.

**Table 2:** RNA oligonucleotide staining of human pancreases

Sample Group	ID	Age	Sex	Fixation	Viral RNA +*	Islet viral RNA +
VP1+ T1D	**E560**	42	F	BF	**++++**	**+**
VP1+ T1D	**11746**	6	M	HgCl_2_	**++**	**+**
VP1+ T1D	**E375**	11	F	FS	**+**	**+**
VP1+ T1D	**E554A**	7	M	Bouin	**- §**	**+**
VP1 – T1D	**E428**	5	M	BF	**++**	**+**
VP1 – T1D	**E514**	23	M	BF	**-**	**+**
VP1 – T1D	**E235**	6	M	HgCl_2_	**-**	**- §**
VP1 – T1D	**8869**	8	M	HgCl_2_	**+**	**- §**
Non-diabetic control	**8579**	7	-	HgCl_2_	**-**	**-**
Non-diabetic control	**12054**	7	-	HgCl_2_	**-**	**-**
Non-diabetic control	**330/71**	47	M	BF	**-**	**-**
Non-diabetic control	**21/89**	4	F	BF	**++**	**-**
Non-diabetic control	**274/91**	6	M	BF	**-**	**-**
Non-diabetic control	**191/67**	25	M	BF	**-**	**-**
Non-diabetic control	**315/89**	9	M	BF	**+**	**+**
Non-diabetic control	**540/91**	11	M	BF	**-**	**-**

Summary of the comparative study of pancreata from T1D and non-diabetic control patients. Sections were first stained for viral RNA, with a combination RNA probe sets CVB_1, CVB_2 and CVB_3, followed by insulin staining. - = less than 10 fully-infected cells, + = 10 to 30 fully-infected cells, ++ = 30 to 100 fully-infected cells, +++ = 100 to 200 fully-infected cells, ++++ = more than 200 fully-infected cells in the exocrine. ^§^E554A showed ten fully-infected cells, E235 contained just 5 islets on the section and 8869 just 2 full and 1 fragmented islets (very small sections).

## DISCUSSION

In this study we present a robust method to identify and localize enteroviral RNA in FFPE tissue, which was established for the analysis of viral infection in pancreata from patients with T1D. The use of RNA probes allowed the discrimination of virally-encoded RNA from virus replication loci with high fidelity and sensitivity. Adapting a method from Raj et al. [28, 29], we created a set of short fluorescently labeled oligonucleotide probes that anneal to common regions of the coxsackievirus family *in situ* to target single RNA molecules.

Probe sets are easily developed using the online Stellaris^®^ RNA FISH Probe Designer. With three different sets of probes we were able to localize viral RNA in all tested tissue including FFPE tissues from patients with proven coxsackievirus infection and in the pancreas of 7 of 8 patients with T1D.

Use of our probe sets with the method described can be routinely applied for virus identification in the pancreas (and, in principle, in any other tissue). It should also now be possible, to increase the specificity of the probe sets for particular virus strains in order to specifically characterize the infection of pancreatic islets in patients with T1D. This is a major undertaking, but is possible using the well-preserved and -characterized FFPE tissues available within the nPOD initiative.

Although enteroviruses (including members of the Coxsackie B virus/ CVB family), have been suspected as potential triggers of T1D for a long time, firm evidence of viral genome expression in the pancreas has been largely elusive.

Most evidence supporting a causative role for enteroviral infections in diabetes onset have come from epidemiological studies [4–6, 11] or from the identification of viral antigens in fixed pancreatic tissue [33, 34]. By contrast, there have been few successful attempts to detect viral genome, except in isolated islet samples or in a small number of samples where positive signals were seen by in situ hybridisation.

For a long time the approach to identify the disease's trigger has been based on the four Koch's postulates [35], which were formulated only considering acute infection but are unfitted when the infection has been encountered long before manifestation of the disease. From tail biopsies of living newly diagnosed T1D patients of the DiViD study (3-9 weeks after T1D onset), VP1 was detected in the pancreas in all patients in 1.7% of the islets. This 100% association of VP1 and T1D could lie in the VP1 characteristics, which is highly expressed at acute viral infection and diminishes in persistent infection [17]. Indirect evidence has been provided to confirm islet cell enteroviral infection by the demonstration of human leukocyte antigen (HLA) class I expression in all patients. In contrast to HLA class I and VP1, viral genome was found in less; 4 of 6 patients at very low concentrations (by PCR, >40 cycles). Although this was a very small study, it was the first showing full correlation of viral RNA by at least one method [17] and convinces that a pancreatic enterovirus infection occurs before or at the time of T1D onset and may be causative for auto-immune activation and β-cell destruction. Nevertheless, the DiViD study also, suggests no evidence of acute infection, and thus confirms earlier studies from the UK and nPOD cohorts pointing at chronic, low-grade infection [34].

Several hypotheses have been proposed to explain why the viral genome has proven so difficult to detect in the islets of patients with T1D [1]: (i) viral infection could act as an initial trigger to activate autoimmunity but the infection may not sustain for long periods; one or multiple viruses can act in concert or in waves leading slowly to β-cell failure. (ii) The virus genome may be modified such that it becomes persistent with very low copy numbers present in infected cells being then difficult to detect [1]. In this context, coxsackieviruses have been shown to establish persistent infections in human heart [22] and in the mouse pancreas [36] via a process involving deletion of the 5′-UTR of the viral genome [14], viruses would be undetected by classical PCR/VP1 staining approaches. Whether this happens in human pancreas has been yet not verified.

In the present work, we have overcome these difficulties by deploying our highly sensitive FISH method to provide evidence that enteroviral genome can be detected more frequently in the pancreas of patients with T1D than in equivalent controls.

The technique applied in this study combines the advantages associated with access to FFPE samples (which retain tissue morphology and allow precise localization of cellular and tissue structures) with a highly sensitive detection method. The sensitivity of detection was much greater than that achieved with classical immunohistochemistry to detect the viral capsid protein, VP1, implying that it is ideal for use in circumstances where viral replication may be compromised [36].

To verify these conclusions, we compared directly results obtained by RNA-FISH with those employing a widely used anti-VP1 (clone 5-D8/1) antibody in infected FFPE material. We carefully established optimized conditions for specific virus detection to avoid interactions of VP1 antibody with other cellular proteins. As a result, we were able to identify viral RNA with much greater sensitivity such that a single RNA molecule was labelled. The use of other sensitive techniques, which might match the sensitivity achieved here (such as PCR, either direct or nested), is limited because of the low accessibility and stability of nucleic acids [26]. By contrast, improvements in *in situ* hybridization (ISH) and fluorescent *in situ* hybridization (FISH) have increased both the sensitivity and specificity of RNA detection such that they now represent a valid alternative to PCR, with the advantage that they can spatially localize particular RNA molecules within fixed cells [25].

The unique advantage of the small contiguous probe sets presented in this study is that RNA duplexes are detected via microscopy, because of direct conjugation of fluorophores to the probe [29]. In order to obtain a detectable signal, multiple probes must then bind to their target RNA to allow the close apposition of sufficient fluorophores for detection under the microscope. A key advantage of this approach is that the potential “off-target” binding of a few oligonucleotides from the probe pool will be either undetectable or where it is detected, can be readily distinguished from the much brighter spots that correspond to the true targets. A minimum of 17 out of 40 probes must bind in order to generate a specific signal in accordance with the Abbe diffraction limit [29].

A significant problem in fluorescence microscopy can be a high background signal, generated either intrinsically from cellular molecules or induced by the fixatives [37, 38]. This high background often precludes accurate analysis of immunostained pancreatic islets in FFPE tissue and is also enhanced by their high auto-fluorescence [39]. The interference from natural fluorescence relates to the tissue type and is due to the presence of endogenous molecules such as flavins, lipofuscins, reticulins, reduced NADP(H), collagen, elastin [37]. Pancreatic tissue has low accretion of flavins and lipofuscins [38], yet the fixatives used to preserve tissue morphology could increase signal background. FFPE samples are highly susceptible to this because of wax crystals and formaldehyde forming covalent bonds with adjacent amino-groups via through Schiff reactions. This can result in an intense fluorescent background with a typical emission between 450 and 650 nm [37, 38]. Here, we refined the de-waxing and hybridization conditions to decrease the background fluorescence and to enhance signal detection.

Of note, our modified deparaffinization/ hybridization protocol had no effect on either tissue morphology or the signal intensity at different wavelengths as shown, for example, by the strongly retained DAPI staining.

Despite the labour-intensive pre-hybridisation protocol employed to ensure background signal reduction, the use of small contiguous RNA-labelled oligonucleotide probes offers several advantages when compared to either classical FISH (using long probes of >100nt) or indirect FISH that relies on intense signal amplification. Firstly, the strength of the bonds between the probe and the target is influenced by various factors such as formamide, salt concentration, temperature and pH. All these factors are easier to control for short than for long sequences. Secondly, systems which use branched probes for signal amplification may be more influenced by RNA degradation, because of the reduced freedom in positioning and flexibility that is allowed for the docking of two consecutive probes.

Sensitivity and specificity of our probe set was tested on different virus strains as well as on various embedding procedures and was compared with indirect FISH on a set of previously analysed samples [25] as well as on newly prepared samples [30]. Our viral probes showed a sensitivity comparable to that of PCR analysis of cell lines when tested in infected cell lines.

Probe specificity was further tested in cell culture conditions and on a cell array [25]. We were able to successfully detect all the viruses with a probe-sequence homology of greater than 60%. From the alignment of our CVB_1 set probes with CVA5 and HPeV1, only 11/40 and 0/40 oligos showed a perfect match and, as expected, these viruses were not detectable by our probe set.

As mentioned above, with the use of small contiguous RNA probes we were able to overcome the issue of RNA stability/degradation in samples, which had been stored over long periods of time. In particular, no signal loss occurred in samples processed at various points over a time course of 3 years.

To test the utility of our probes in archive pancreatic tissue we undertook a blinded evaluation of T1D pancreata from a UK [40, 41] cohort, and expanded our CVB_1 probe set with the addition of 2 complementary sets, namely CVB_2 and CVB_3. This broadened the opportunity to detect enteroviruses across the entire group B when used in combination. By this approach, we detected viral RNA in 7 of 8 T1D pancreata; 4 of which had also given positive signals using immunohistochemical analysis of VP1 expression.

Among the group of 7 RNA positive individuals, 6 had viral RNA in insulin-containing cells whereas only 4 were also immune-positive for VP1 (the parameter used to define “T1D VP1+” in this concordance study). The two pancreata, which were negative for viral RNA within islets using FISH probes were also immunonegative for enteroviral VP1. Typically, 1-2 islets per section showed viral genome staining but, unlike immunoreactive VP1, enteroviral RNA was not restricted solely to insulin positive cells.

Viral RNA was detected in two young non-diabetic control pancreata but at a much lower prevalence than among patients with T1D. Thus, enteroviral infection may not be absolutely unique to the islets of patients with T1D and it remains possible that cellular response to viral infection (rather than infection per se) is a critical determinant of autoimmunity.

In conclusion, we have developed a stringent and highly specific method for the detection of enteroviral genome in fixed pancreatic tissue with high sensitivity. We have used this method to confirm that viral RNA is detected in the pancreas in 7 of 8 patients with T1D and in only 2 of 8 controls. We propose that this new method may find wide applicability in future studies of the viral aetiology of type 1 diabetes.

## MATERIALS AND METHODS

### Cell culture and viruses

Human islets were isolated and cultured as previously described [42], HEK 293 and FHRK-4 were cultured in Dulbecco's Modified Eagle's Medium (DMEM) at 5.5 mM glucose, CM9 and INS-1E in RPMI-1640 (Lonza) at 11.1 mM glucose. All media were supplemented with 1% penicillin/streptomycin and 10% fetal calf serum (FCS) or 1% FCS (FHRK-4). All cellular experiments were performed at 37°C and 5% CO_2_, if not stated otherwise.

All viruses were propagated in FHRK-4 cells and purified in a sucrose gradient (40% sucrose, 10mM Tris pH 7,5, 100mM NaCl and 1mM EDTA), aliquots were stored at -80°C as previously described [43]. Cells were grown to confluency of 80-90% and infected with multiplicity of infection (MOI) of 5 supplemented growth media without FCS. Virus-containing media was replaced with 1% FCS fresh media after 2h of infection (post-infection).

### Probe design

Premade Stellaris^®^ FISH Probes recognizing human GAPDH labeled with Quasar 570 (Catalog #SMF-2026-1-BS) and custom Stellaris^®^ FISH Probes, each recognizing various enteroviral strains, labeled with Quasar 570 were purchased from Biosearch Technologies, Inc. (Petaluma, CA). Briefly, the initial set of custom RNA FISH oligonucleotides (CVB_1) was designed on the CVB3 consensus based sequence (M33854.1). The consensus sequence was generated from a ClustalW alignment of several coxsackieviruses to localize conserved regions, with thermodynamic characteristics fitted to the probe design [26]; in total, 50 regions of about 20 nucleotides (nts) in length with 2 nucleotides gap between adjacent regions and 45% GC content. Suitability of the regions for probe design were then verified using the Stellaris^®^ RNA FISH Probe Designer (Biosearch Technologies, Inc., Petaluma, CA) available online at www.biosearchtech.com/stellarisdesigner As a result, 40 probes (Figure S4) distributed throughout the whole target genome were generated. For the CVB_2 and _3 probe sets design, 106 genome sequences of viruses belonging to the enterovirus group B family were aligned (Figure S6) and divided into three subgroups based on sequence similarities. For each subgroup probes were designed based on newly generated consensus sequences as described before [26]. From the pool, 35 and 36 oligo probes were selected as CVB_2 and -3 pool for a sequential combinatorial approach to detect all the members of the enterovirus group B.

### RNA FISH in FFPE tissue samples

The pre- and post-hybridization protocol for RNA fluorescent in situ hybridization (FISH) was modified based on the manufacturer's instructions available online at www.biosearchtech.com/stellarisprotocols (version 11.04.2013).

We adapted the pre-hybridization procedure by increasing the temperature of the xylene bath, in order to also access older FFPE tissue libraries [41]. The melting temperature for low-melting point paraffin is around 52°C and for normal paraffin is around 65-70°C [44, 45]. Melted paraffin can remain on the section forming wax crystals at lower temperatures (~57°C). Residual paraffin particles and wax crystals from the embedding procedure were removed by a series of Xylene washes (20 min at 70°C; 10 min at 70°C; 10 min at room temperature), followed by rehydration in an ethanol (EtOH) gradient (100%, 100% and 95%) for 10 min each and for 1 h in 70% EtOH at room temperature. Finally, sections were rinsed twice with H_2_O for 1 min. All steps were performed with constant stirring.

Slides fixed with HgCl_2_ where washed with iodine to remove mercury, before the deparaffinization protocol. To facilitate probe annealing, sections were incubated for 20 min in 0.2M HCl (room temperature) and washed with 2x SSC Buffer (15 min, 70°C, slightly shaking) and PBS (2x 1 min, room temperature). Pepsin (Sigma) was applied and washed off after 10 min incubation at 37°C with PBS (2 times 1 min, room temperature). To quench any remaining autofluorescence of biological molecules 0.5-1% Sudan Black (Sigma-Aldrich) in 70% EtOH was added for 20 min (room temperature) and thoroughly washed off with PBS (3x times 5 min, room temperature). Tissues were equilibrated with washing buffer (10% formamide, 2xSSC, 2x, 5 min at room temperature) before hybridization. Stellaris^®^ FISH Probes (GAPDH 0,125 µM; viral RNA 0,25 µM) were diluted in hybridization buffer (10% w/v Dextran sulfate, 10% formamide, 2xSSC) and samples incubated overnight at 37°C in a humidified chamber. Hybridization mix was aspirated and sections were washed extensively. Stringency was increased with each washing step to remove any unspecific probe binding, thus reducing background noise and increasing relative signal intensity. Slides were washed at 37°C with constant agitation; twice with 2xSSC and 10% formamide for 20 min, twice with 2xSSC for 15 min, twice with 1xSSC for 15 min, once with 0.1x SCC for 15 min and 5 min. VECTASHIELD^®^ antifade mounting medium (Vector laboratories) including 4′,6-Diamindino-2-phenylindole (DAPI) was immediately added and images were acquired with a Nikon Ti MEA53200 (NIKON GmbH, Düsseldorf, Germany) microscope. A 60x oil-immersion objective (N.A. 1,4). TRITC filter (ex. 520- 560nm) was used to acquire images of the Quasar 570 labeled probes. Control images were always taken with the FITC filter (ex. 465- 495nm) at the same exposure time to ensure no false-positive signals caused by a bleed-through from one channel to another. NIS-Elements BR (NIKON GmbH, Düsseldorf, Germany) and ImageJ (NIH, USA) were used for image analysis.

### RNA FISH in cell lines

Cells were cultivated on 13×13 mm #1 microscope cover glasses (Marienfeld GmbH & Co. KG, Lauda-Königshofen, Germany) in 24-well plates to a confluency of 80-90% and treated as indicated. Further processing followed manufacturer's instructions available online at www.biosearchtech.com/stellarisprotocols (11.04.2013). Briefly, cover glasses were rinsed with PBS and cells were fixed (3,7% formaldehyde, 1x PBS) for 10 min at room temperature, followed by two washes with PBS (room temperature). To permeabilize the cellular membrane 70% EtOH was added for overnight incubation at 4°C. EtOH was removed, cells were equilibrated with washing Buffer (5 min, room temperature) and respective RNA FISH probe mix was added for overnight hybridization at 37°C in a humidified chamber. Hybridization mix was aspirated; cells were washed twice with washing buffer (20 min, 37°C) and rinsed with 2xSSC (room temperature). Cover glasses were placed on glass slides with VECTASHIELD^®^ antifade mounting medium (Vector laboratories) including DAPI and analyzed as described above.

### Immunohistochemistry

FFPE sections were deparaffinized as described above. Antigen retrieval was performed in heated unmasking solution (Vector laboratories) in three consecutive 5 min microwave cycles (full power). Sections were blocked (TBS, 3% BSA IgG free, 0.2% Tween) for 1h at room temperature and primary anti-VP1 (DAKO, clone 5-D8/1, dilution 1/2000) was applied for over night incubation at 4°C. FITC anti-mouse (1/100) for 1h at room temperature was used as secondary antibody.

### Co-immunostaining of insulin and RNA-FISH

Samples were first probed for RNA targets and analyzed as described above. Afterwards, the coverslip was gently removed, slides were immersed in washing buffer and anti-insulin (DAKO) was applied for overnight incubation at 4°C. Sections were washed three times with washing buffer (5 min, room temperature), incubated for 1h at room temperature with fluorescein isothiocyanate (FITC) anti-guinea pig, were washed 3x with 2xSSC (5 min, room temperature) and mounted with VECTASHIELD^®^ antifade mounting medium (Vector laboratories) including DAPI.

### Co-immunostaining of VP1 and RNA-FISH

Cells were infected with CVB3 (MOI 1000) for 1h (16°C). Media was changed and temperature shifted to 37°C. Cells were fixed 30 min post-infection using a solution of 3,7% formaldehyde in 1x PBS for 10 min. Cells were permeabilized with 70% EtOH overnight at 4°C and probed with RNA probes as described above. Cells were washed and stained for VP1 (1/2000; 1h; RT) and FITC anti-mouse as secondary antibody.

### RNA isolation, reverse transcription and real time PCR

RNA was extracted using Trizol^®^ reagent (Invitrogen, Darmstadt, Germany) according to manufacturer's protocol. Extracted RNA was treated with DNAse I (Ferments Life Science, Waltham, MA, USA) and reverse transcribed with ReverseAid kit (Fermentas) according to manufacturer's instructions. Real time PCRs reactions were prepared according to Applied Biosystems guidelines using SybrGreen assay and performed in a StepOnePlus instrument (Applied Biosystems, Carlsbad, CA, USA). PCR efficiencies were monitored for each sample according to a previously described approach [46]. Results are presented as relative quantification. SybrGreen Primers used:

CVB fw 5′ GGCCCCTGAATGCGGCTAAT 3′,

CVB rev 5′ TGGCTGCTTATGGTGACAATTG 3′;

Microglobulin β2 fw 5′ TTTACTCACGTCATCCAGCAG A 3′;

Microglobulin β2 rev 5′ CGGCAGGCATACTCATCTTT 3′.

RNAse treatment

CVB3 infected FFPE human islets sections were prepared as described above. To digest any viral or endogenous RNA 100µg/ml RNAse A (Macherey-Nagel) in 2xSSC buffer or 2xSSC Buffer alone was applied to the sections for 1h at 37°C. At this salt-concentration (150mM NaCl) single-stranded, as well as double-stranded RNA, is enzymatically cleaved by RNase A [47].

### Viral dilution and FISH analysis

CVB 3 was diluted in RPMI-1640 media in logarithmic steps, starting from 10^2^ (MOI 100) to 10^-8. Infection media was added to respective cells in a 24-well plate either grown on coverslips or without. Cells were directly transferred to a tabletop centrifuge and spun for 1h at 16°C and 105 rcf., followed by 1.5h (HEK 293) or 3h (CM9) post-infection at 37°C. Afterwards one set of cells was used for RNA isolation and the other for microscopical analysis. Ten images were acquired for each condition, choosing the same areas of the slide for every dilution.

## SUPPLEMENTARY FIGURES AND TABLES


