# Supplementary material for: Detection and localization of viral infection in the pancreas of patients with type 1 diabetes using short fluorescently-labelled oligonucleotide probes
Source: Oncotarget. 2017 Jan 29;8(8):12620–36. doi: 10.18632/oncotarget.14896 (PMC5355040; doi:10.18632/oncotarget.14896)
Supplement: Supplementary file 1 [file oncotarget-08-12620-s001.pdf]

Detection and localization of viral infection in the pancreas of patients with type 1 diabetes using short fluorescently-labelled oligonucleotide probes

Supplementary Material

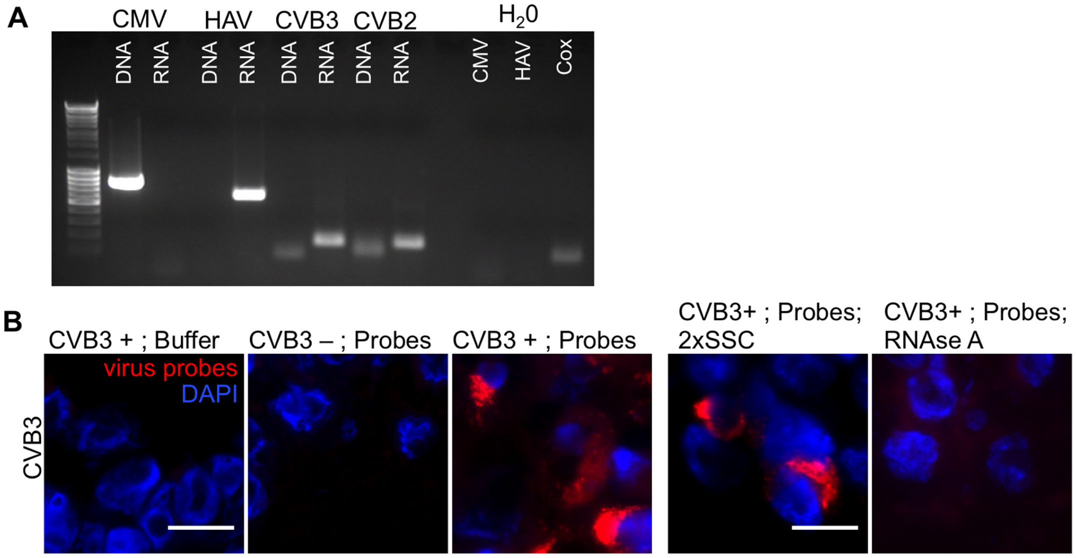

Figure S1. Specificity of viral RNA probes
(A) Confirmation of viral-infection in the probe sensitivity test of Fig.2. DNA and RNA of CMV, HAV, CVB3 and CVB2 were isolated and analyzed for the presence of viral genome. Each sample was processed in parallel with or without DNAse digestion. (B) CVB3 infected or uninfected FFPE human islets were either stained with buffer or CVB_1 oligos (left panel) or treated with 100ug/ml RNAse A or 2xSSC Buffer (control) for 1h at 37°C before hybridization with RNA FISH probes (right panel). Nuclei were stained with DAPI (blue); scale bar depicts 10µm.

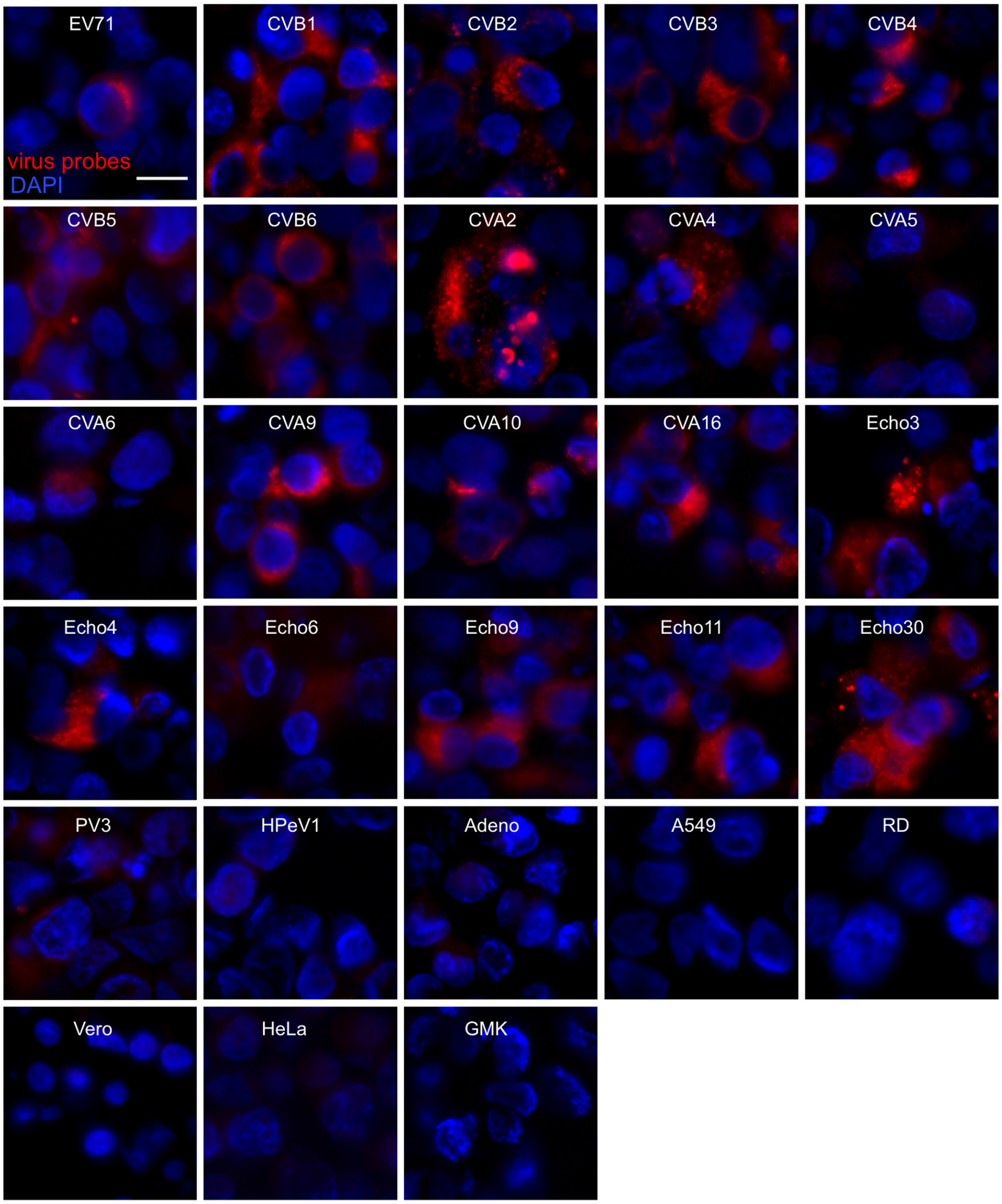

Figure S2. Summary of custom RNA oligonucleotide staining of different picornaviridae and control viruses. **Representative images of the viral RNA (red) staining on the cell array shown in Table 1.** Nuclei were stained with DAPI (blue), scale bar depicts 10µm.

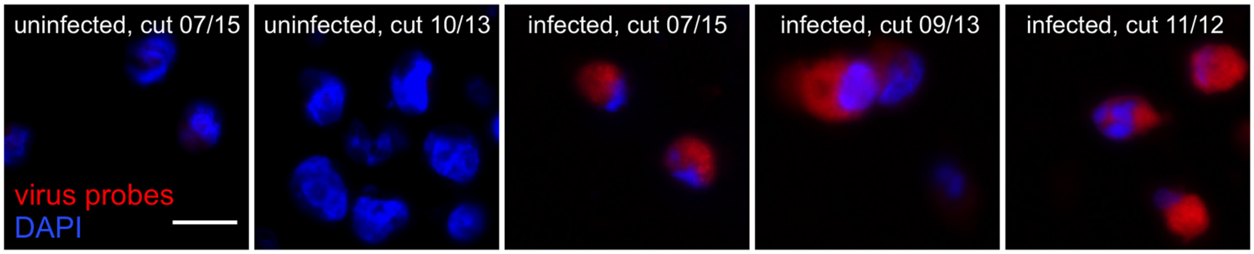

Figure S3. RNA-oligonucleotide labelling is consistent despite sample conditions
GMK cells FFPE sections of different age from non-infected (2015 and 2013) and CVB1-infected (2015, 2013 and 2012) were probed for viral RNA (red). Nuclei were stained with DAPI (blue); scale bar depicts 10µm.

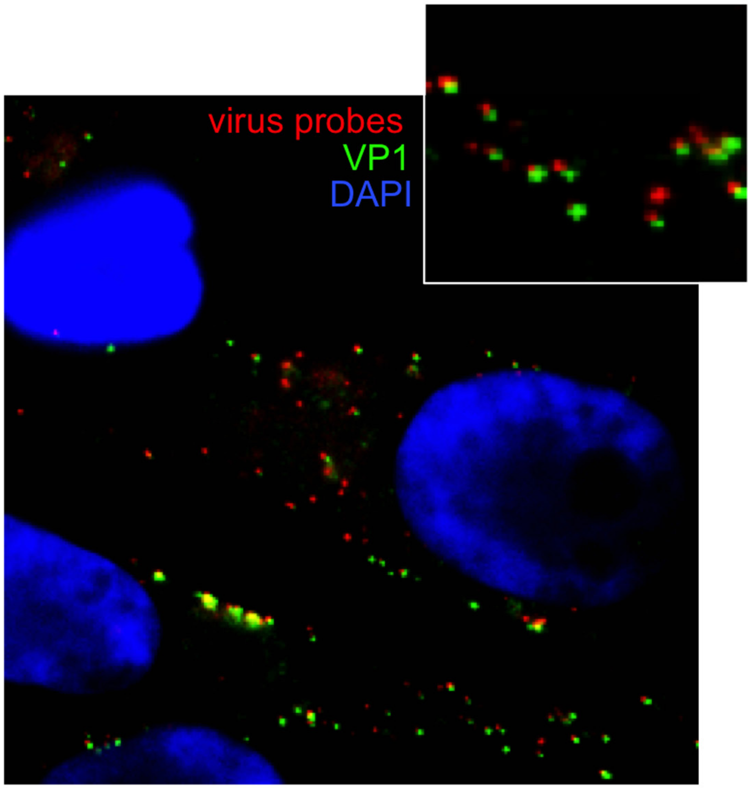

Figure S4. Coupling RNA-oligonucleotide labelling and immunohistochemistry
Magnified and enhanced image from Fig.4B, middle panel showing colocalization of VP1 (green) and viral RNA oligonucleotide probes (red).

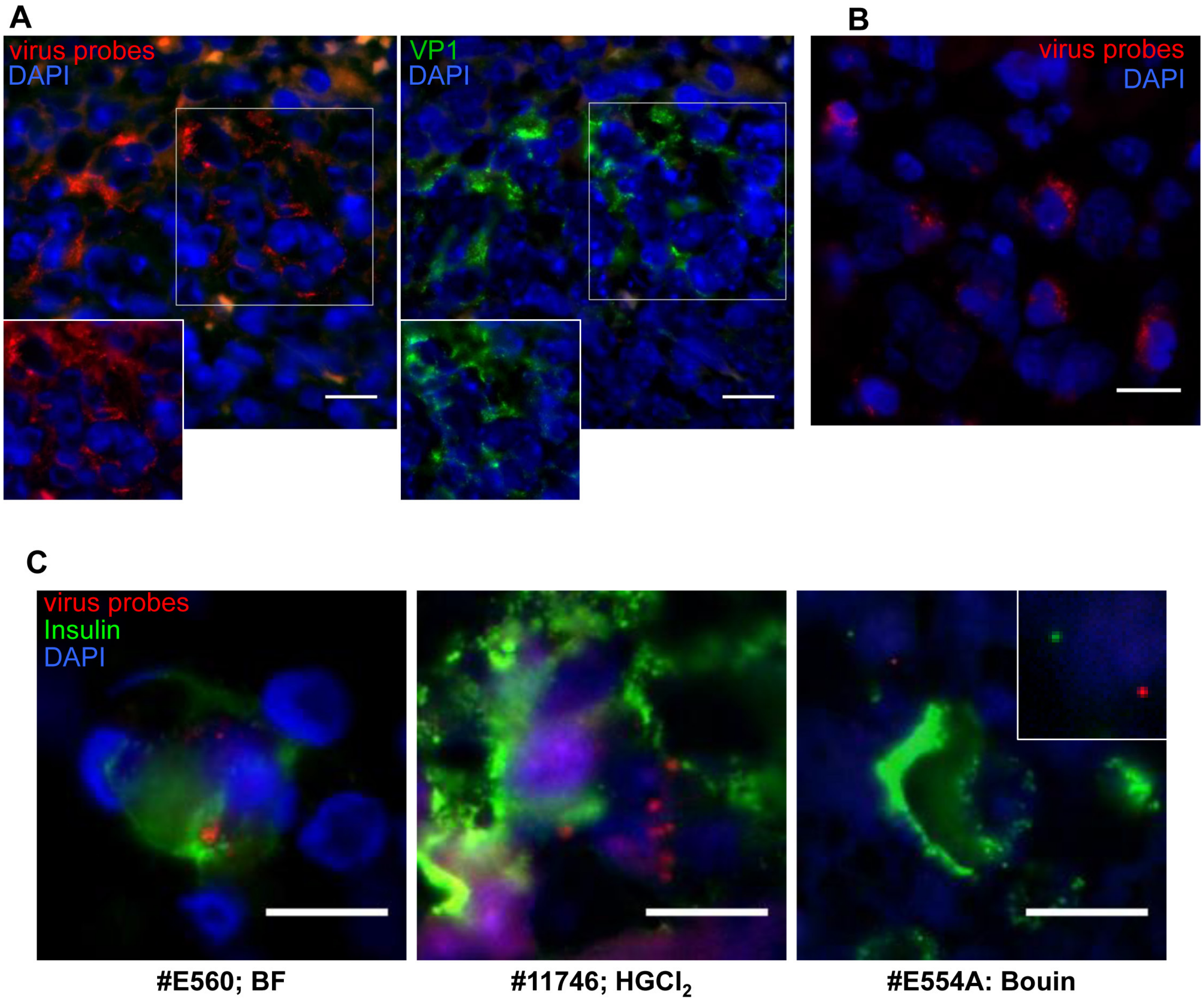

Figure S5. Custom viral RNA oligonucleotides bind tissue- and fixative-independent
(A) Detection of viral RNA (red) and viral protein 1 (VP1) (green) in the same region of an FFPE spleen sample. Tissue was first stained for viral RNA, analyzed and probed for VP1. (B) CVB-infected neonatal heart FFPE section was tested positive for viral RNA with fluorescent-labeled oligonucleotides (red). (C) RNA-FISH probes perform independent of the fixative used in human pancreatic sections as shown in the concordance study (Table 3). Nuclei were stained with DAPI (blue); scale bar depicts 10μm.

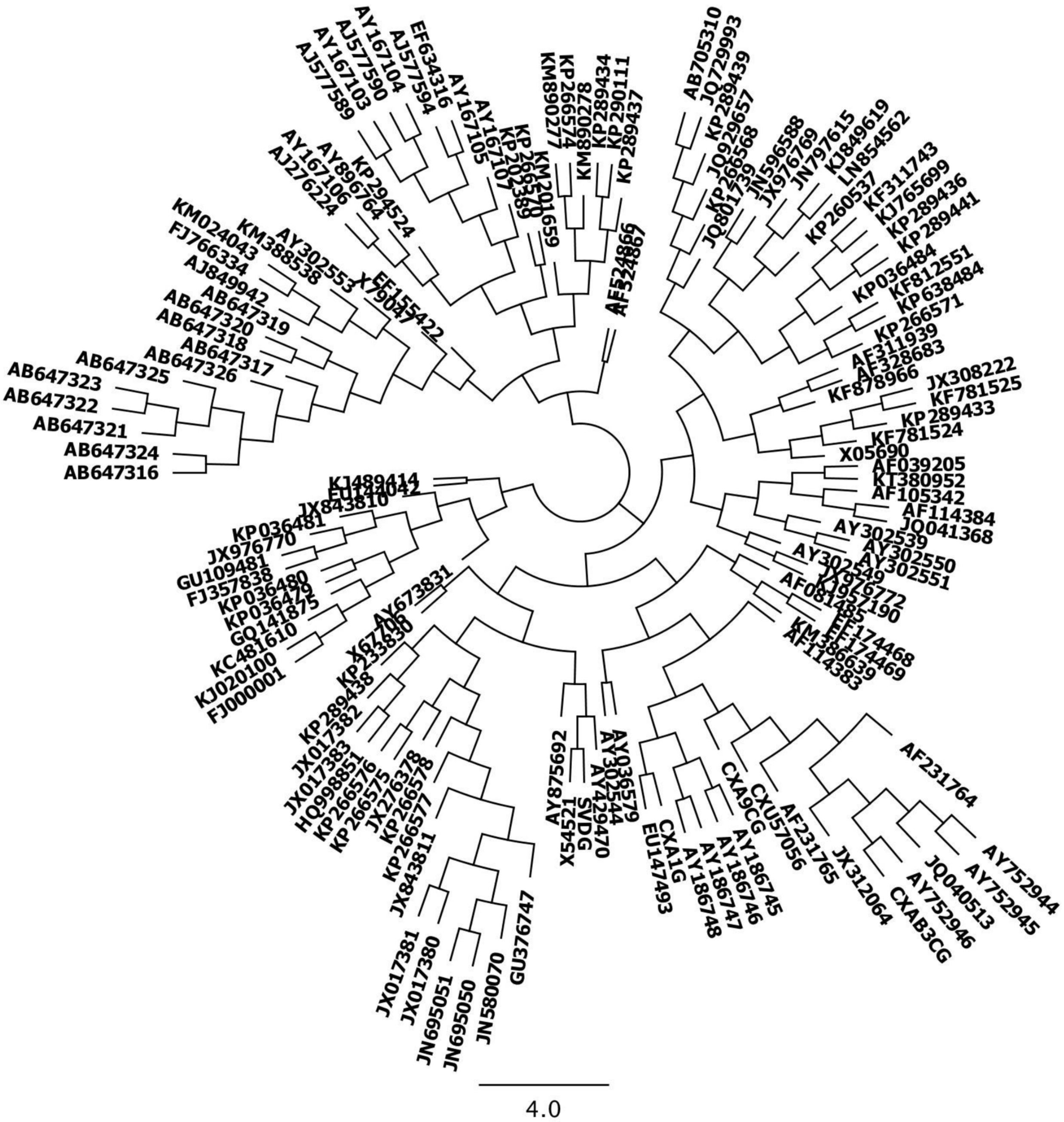

Figure S6. Genome alignment.

Enterovirus genome alignment for the design of CVB_2 and CVB_3 probe sets (Geneious version (9.1.5) (http://www.geneious.com) [48]).

Table S1: Counted fluorescent spots of RNA-FISH sensitivity test

Dilution	HEK293 (total spots)	CM9 (total spots)	HEK293 (dots/cell)	CM9 (dots/cell)
10^{2}	7378	387	19,80656173	0,525101759
10^{1}	1025	161	2,282483251	0,259821157
10^{0}	98	103	0,289588584	0,130535155
10^{-1}	51	39	0,112774084	0,058245893
10^{-2}	39	20	0,096695942	0,025043732
10^{-3}	18	33	0,033731891	0,048228387
10^{-4}	8	16	0,02117113	0,022302297
10^{-5}	8	10	0,017853038	0,014837777
10^{-6}	14	23	0,036317252	0,029477528
10^{-7}	6	20	0,018873785	0,028428992
10^{-8}	9	19	0,022544999	0,026538136

CM 9 and HEK 293 were infected with a dilution series (MOI 10^{2}–10^{-8}) of CVB3 and stained with custom-designed FISH probes (Fig.3). Ten single images were acquired for each dilution and single fluorescent spots as well as number of cells were manually counted (ImageJ).

Table S2: CT values of CVB and housekeeping genes

	CM CVB		CM HK		HEK 293 CVB		HEK 293 HK	
MOI	C_T Mean	C_T SD	C_T Mean	C_T SD	C_T Mean	C_T SD	C_T Mean	C_T SD
10^{2}	19,1	± 0.2	23.2	± 0.1	18.8	± 0.1	20.4	± 0.1
10^{1}	22,2	± 0.0	23.4	± 0.0	22.9	± 0.0	21.1	± 0.0
10^{0}	26,3	± 0.5	23.2	± 0.1	25.7	± 0.2	21.2	± 0.0
10^{-1}	29,7	± 2.0	22.9	± 0.0	27.8	± 0.2	21.4	± 0.0
10^{-2}	35,0	± 1.9	23.8	± 0.0	30.7	± 0.5	22.5	± 0.0
10^{-3}	33,9	± 0.7	23.7	± 0.1	29.0	± 0.2	21.0	± 0.0
10^{-4}	34,9	± 2.1	23.1	± 0.0	30.4	± 0.5	21.7	± 0.0
10^{-5}	33,4	± 1.7	22.6	± 0.0	31.1	± 0.6	21.4	± 0.0
10^{-6}	31,5	± 2.0	23.3	± 0.0	31.9	± 0.5	21.6	± 0.9
10^{-7}	28,5	± 1.9	22.6	± 0.1	37.2	± 1.8	23.2	± 0.1
10^{-8}	37,5	± 0.6	23.8	± 0.1	32.9	± 1.5	20.7	± 0.0

CM9 and HEK 293 were infected with a dilution series (MOI 10^{2}–10^{-8}) of CVB3. Viral RNA was extracted and analyzed with PCR (Fig.3). Shown are the CT and SD (standard derivation) values for the respective viral RNA (CVB) and housekeeping gene (HK, β2-Microglobulin).

Table S3: CVB_1 set sequences

#	Probe 5'→ 3'	Sequence 5' → 3'
1	caacccacaggctgttttaa	ttaaaacagcctgtgggttg
2	aacaggcgcacaaaggtacc	ggtacctttgtgcgcctgtt
3	ctattgatactcagtccggg	cccggactgagtatcaatag
4	taacgaacgctttctccttc	gaaggagaaagcgttcgtta
5	gtagtgctgagcgaaacact	agtgtttcgctcagcactac
6	tgactcatcgacctgatcta	tagatcaggtcgatgagtca
7	caccatgtctgtattagagc	gctctaatacagacatggtg
8	aggactaccaactagctcaa	ttgagctagttggtagtcct
9	ttaggattagccgcattcag	ctgaatgcggctaatcctaa
10	gaaacacggacacccaaagt	actttgggtgtccgtgtttc
11	caattgtcaccataagcagc	gctgcttatggtgacaattg
12	ggccaatccaatagctatat	atatagctattggattggcc
13	gtagatgtttgccacacgta	tacgtgtggcaaacatctac
14	tgttccattgcatcatcttc	gaagatgatgcaatggaaca
15	agtgattctttcaggaggtt	aacctcctgaaagaatcact
16	gcgtactttctgcaatagtg	cactattgcagaaagtacgc
17	ctgtatgtaattgctcatct	agatgagcaattacatacag
18	ggttcaatacggcatttgga	tccaaatgccgtattgaacc
19	ggcatagatcgtccataatc	gattatggacgatctatgcc
20	gaaaccatttggcagaacaa	ttgttctgccaaatggtttc
21	gacatgggcatgtttatctt	aagataaacatgcccatgtc
22	gacatgtttctcaatttgga	tccaaattgagaaacatgtc
23	ctgaacttctcattccggtt	aaccggaatgagaagttcag
24	tcattaacctccacttcctc	gaggaagtggaggttaatga
25	tgccacctaggtttaggaag	cttcctaaacctaggtggca
26	gttgtacataagcattctct	agagaatgcttatgtacaac
27	cttggtgtgttgatgactgg	ccagtcatcaacacaccaag
28	tgtgtgttgacatttcctat	ataggaaatgtcaacacaca
29	tccagtttcattggttcagt	actgaaccaatgaaactgga
30	catacttgtccatacattcc	ggaatgtatggacaagtatg
31	gtcaatgtagtttgtctctt	aagagacaaactacattgac
32	caagtcaatccctttgtaca	tgtacaaagggattgacttg
33	catcaccatatgcgatcatc	gatgatcgcatatggtgatg
34	gtactgttcatctgctctaa	ttagagcagatgaacagtac
35	tcgtgtatgtctttcatggg	cccatgaaagacatacacga
36	atcttgggtgttctttggat	atccaaagaacacccaagat
37	tgaactcctcatattcgtgc	gcacgaatatgaggagttca
38	agtagggttaagccaatcta	tagattggcttaaccctact
39	ccgttatctggttcggttag	ctaaccgaaccagataacgg
40	cgaatgcggagaatttaccc	gggtaaattctccgcattcg

Sequences of probes and respective viral target regions of set CVB_1. Designed with Stellaris® RNA FISH Probe Designer (Biosearch Technologies, Inc., Petaluma, CA).

Table S4: CVB_2 set sequences

#	Probe 5'→ 3'	Sequence 5' → 3'
**1**	aggtttctcgaagtaattgg	ccaattacttcgagaaacct
**2**	tgatctacactggggttgtg	cacaaccccagtgtagatca
**3**	ttacgacagactgcccactg	cagtgggcagtctgtcgtaa
**4**	caaagtagtcggttccgctg	cagcggaaccgactactttg
**5**	tgcttaccattgtcactgtt	aacagtgacaatggtaagca
**6**	ccaacatcccatatgacgtg	cacgtcatatgggatgttgg
**7**	acatgagctttgcacatcag	ctgatgtgcaaagctcatgt
**8**	acggcatttggacttgaact	agttcaagtccaaatgccgt
**9**	tcagtccggggtaacagaag	cttctgttaccccggactga
**10**	gtttctcgaagtaattggcc	ggccaattacttcgagaaac
**11**	tagctcaatagactcttcgc	gcgaagagtctattgagcta
**12**	ctgctccgcagttaggatta	taatcctaactgcggagcag
**13**	agcagccagttcaagaataa	ttattcttgaactggctgct
**14**	ggccaatccaatagctatat	atatagctattggattggcc
**15**	tgtgtggttattgtggagtt	aactccacaataaccacaca
**16**	gttgaagggaatgcctgacc	ggtcaggcattcccttcaac
**17**	gggttgtgctagtaaactca	tgagtttactagcacaaccc
**18**	tgtatggcatcactatggtg	caccatagtgatgccataca
**19**	tgtgaagttgtagtgcctaa	ttaggcactacaacttcaca
**20**	aaattgcgtactccctggtg	caccagggagtacgcaattt
**21**	cctggtatattcatactagg	cctagtatgaatataccagg
**22**	cacaacggagtccacttctg	cagaagtggactccgttgtg
**23**	aacatttccatttggtcggt	accgaccaaatggaaatgtt
**24**	ggcattaatggtcactggaa	ttccagtgaccattaatgcc
**25**	acagaaaagtcgttgcaagc	gcttgcaacgacttttctgt
**26**	tttctgagttgctaggtcca	tggacctagcaactcagaaa
**27**	ggactcagaacgagtatggt	accatactcgttctgagtcc
**28**	ctacgcatttgcaccatttg	caaatggtgcaaatgcgtag
**29**	cactggcatgtcttgtgcta	tagcacaagacatgccagtg
**30**	cctcacatatatgtgaccta	taggtcacatatatgtgagg
**31**	gtggttacagtgttaatgtc	gacattaacactgtaaccac
**32**	tggtccctcgaagctaactg	cagttagcttcgagggacca
**33**	gggtagtattcactctcctg	caggagagtgaatactaccc
**34**	aatcccgagccaaaagcgtt	aacgcttttggctcgggatt
**35**	tcatcatggtttctcaccac	gtggtgagaaaccatgatga

Sequences of probes and respective viral target regions of set CVB_2. Designed with Stellaris® RNA FISH Probe Designer (Biosearch Technologies, Inc., Petaluma, CA).

Table S5: CVB_3 probe set sequences

#	Probe 5'→ 3'	Sequence 5' → 3'
1	ggtaccgtgataccagagtg	cactctggtatcacggtacc
2	gggtaacagaagtgcttgat	atcaagcacttctgttaccc
3	tagactcttcgcaccatgtc	gacatggtgcgaagagtcta
4	tgaattcttcatccactgca	tgcagtggatgaagaattca
5	gagccattctataaatttct	agaaatttatagaatggctc
6	acaaaatgcctttctcttct	agaagagaaaggcattttgt
7	acctcgatgttcatatcaaa	tttgatatgaacatcgaggt
8	caacactcttcatcacacgt	acgtgtgatgaagagtgttg
9	tcaagggagtatctgacttg	caagtcagatactcccttga
10	tgaacatctcagttaccagc	gctggtaactgagatgttca
11	ctggtgttaattgctagcac	gtgctagcaattaacaccag
12	cattctcttggtgggtgtac	gtacacccaccaagagaatg
13	gagaagccttgatgaccatt	aatggtcatcaaggcttctc
14	aactcgatttctccttgttc	gaacaaggagaaatcgagtt
15	aggactgctggctctttatt	aataaagagccagcagtcct
16	gccaattgaccagcataatg	cattatgctggtcaattggc
17	tctgcagatctgagttcatc	gatgaactcagatctgcaga
18	aacatcactggtatcttgct	agcaagataccagtgatgtt
19	gctagcgtcatatccagaat	attctggatatgacgctagc
20	ttggtccatctgattgattc	gaatcaatcagatggaccaa
21	cgttgtgccaggccaataag	cttattggcctggcacaacg
22	aaaagagtccaaccacttcc	ggaagtggttggactctttt
23	ccctggatcttgagtgaaat	atttcactcaagatccaggg
24	gagctctgttgccacattac	gtaatgtggcaacagagctc
25	tcgggaaatttccaccacca	tggtggtggaaatttcccga
26	aggtaatggtattgcatgtt	aacatgcaataccattacct
27	cctgtggagttttgggattc	gaatcccaaaactccacagg
28	atggtcagattgccaactc	gagttggcaatctgaccata
29	gacaatcgtcgcactattgt	acaatagtgcgacgattgtc
30	ctgcgatttccatcaagttg	caacttgatggaaatcgcag
31	tcactttgagtgtctgcagg	cctgcagacactcaaagtga
32	tggtttgcatagtgtctgct	agcagacactatgcaaacca
33	aagttgtcaccatcagtacc	ggtactgatggtgacaactt
34	tattgacatacggggtggtg	caccaccccgtatgtcaata
35	gcattgcctatgctgatgaa	ttcatcagcataggcaatgc
36	acggcatttggacttgaact	agttcaagtccaaatgccgt

Sequences of probes and respective viral target regions of set CVB_3. Designed with Stellaris® RNA FISH Probe Designer (Biosearch Technologies, Inc., Petaluma, CA).
